# Eye movements of second language learners when reading spaced and unspaced Chinese texts

**DOI:** 10.3389/fpsyg.2023.783960

**Published:** 2023-03-13

**Authors:** Yaqiong Cui

**Affiliations:** University of Chinese Academy of Sciences, Beijing, China

**Keywords:** interword spacing, eye movements, Chinese as a second language, L2 reading, psycholinguistics and education

## Abstract

Unlike English, Chinese does not have interword spacing in written texts, which poses difficulties for Chinese-as-a-second-language (CSL) learners’ identification of word boundaries and affects their reading comprehension and vocabulary acquisition. The eye-movement literature has suggested that interword spacing is important in alphabetic languages; examining languages that lack interword spaces such as Chinese, thus, may help to inform theoretical accounts of eye-movement control and word identification during reading. Research investigating the interword spacing effect in reading Chinese showed that adding spacing facilitated CSL learners’ reading comprehension and speed as well as vocabulary learning. However, the bulk of this research mainly looked at the learning outcomes (off-line measures), with few studies focusing on L2 learners’ reading processes. Building on this background, this study seeks to provide a descriptive perspective of the eye movements of CSL learners. In this study, 24 CSL learners with intermediate Chinese proficiency were recruited as the experimental group, and 20 Chinese native speakers were recruited as the control group. The EyeLink 1,000 eye tracker was used to record their reading of four segmentation conditions of Chinese texts, namely, no space condition, word-spaced condition, non-word-spaced condition, and pinyin-spaced condition. Results show that: (1) CSL learners with intermediate Chinese proficiency generally spent less time reading Chinese texts with spaces between words, and they showed more gazes and regressions when reading texts without spaces; (2) Non-word-spaced texts and Pinyin-spaced texts interfere with CSL learners’ reading process; and (3) Intermediate CSL learners show consistent eye movement patterns in the normal no-space condition and word-spaced condition. I conclude that word boundary information can effectively guide CSL learners’ eye movement behaviors and eye saccade planning, thus improving reading efficiency.

## Introduction

Learning to read a second language (L2) that is orthographically different from the native language (e.g., native speakers of an European language learning to read Chinese) is challenging. There are three distinct characteristics in the Chinese writing system that make it different from European languages. First, Chinese is a character-based language in which characters occupy the same amount of space in written texts but differ in visual and linguistic complexity (Shen et al., 2012). The majority (approximately 70%) of Chinese words are comprised of two characters, and only a small portion of Chinese words are formed by a single character (approximately 20%) or by three or more characters (approximately 10%; Modern Chinese Frequency Dictionary, 1986). Secondly, there are many homophones in Chinese. In other words, many characters share the same pronunciation but vary in visual forms and meanings. Thirdly, there is no visible interword spacing between words. The lack of spaces between words sometimes poses great difficulties for non-native readers of Chinese because it is difficult to locate the word boundaries given the varying number of characters in Chinese words (Everson, 1986; Bassetti, 2009; Yao, 2011; Blythe et al., 2012; Bai et al., 2013). This characteristic of Chinese, therefore, lends itself to investigating how people read a language that has no spaces between words.

The past few decades have seen a considerable number of studies examining readers’ eye movements when reading alphabetic languages (Rayner, 1979; Epelboim et al., 1994; Rayner and Pollatsek, 1996; Rayner et al., 1998; Perea and Acha, 2009). The main question that these studies have sought to answer is where readers send their eyes while reading and what guides their eye movements.

In terms of the locations where readers send their eyes during reading, Rayner (1979) defined the “preferred viewing location” (PVL) as the locations in a word where the eyes prefer to land while reading and the “optimal viewing position” (OVP) as the initial landing site in a word that results in the shortest gaze durations and fewest refixations. The existence of a PVL has been confirmed in English (Rayner and Pollatsek, 1996; Rayner et al., 1998). Although linguistic factors have been shown to affect a variety of eye movement measures, the fact that the landing sites of the eyes display systematic, word-based patterns suggests that the selection of saccade targets is based on the information of word boundaries, which is provided by the visually salient spaces between words (Rayner, 1998; Rayner et al., 1998). Several studies have shown that removing the interword spaces from alphabetic languages disrupts eye movements (e.g., decreased reading rate and different PVL patterns) and word identification (Rayner and Pollatsek, 1996; Rayner, 1998; Rayner et al., 1998; Perea and Acha, 2009). What can be suggested by these studies is that visible word boundaries may play an important role in helping guide readers’ eye movements during reading. Correspondingly, it may be logical to hypothesize that adding interword spacing to naturally unspaced texts, such as Chinese, may facilitate reading. In particular, this facilitative effect may be more evident for L2 learners of an unspaced language whose native languages are written with interword spacing (e.g., Winskel et al., 2009).

There have been long-held debates on whether interword spacing should be introduced in Chinese written texts, especially in language teaching materials for Chinese learners. While young children and adult L2 learners of Chinese are taught how to read with unspaced texts immediately from the beginning, some parents and teachers have noticed learners’ difficulties with word segmentation and word recognition. Proponents for adding interword spacing, thus, hold that adding interword spacing may make the word boundaries salient for young children and adult L2 learners of Chinese, and thus help them better segment Chinese words, and facilitate their vocabulary learning and reading comprehension (Zhang, 1998). Anecdotal evidence also shows that some L2 learners of Chinese adopt a strategy of manually segmenting words by putting slashes between words when reading Chinese texts. However, others argue that segmenting Chinese words is a linguistically complex task due to a lack of consistent conventions (i.e., of what constitutes a word), which might cause confusion (Yang, 2006; Bassetti, 2009). Building on this background, it is hoped that the results from the current study could contribute to the theoretical and practical aspects of reading a non-alphabetic, character-based language and provide implications for the field of teaching Chinese as a second language (CSL).

### Reading spaced and unspaced texts

A large body of research investigating eye movements when reading naturally unspaced texts has been conducted in Thai, Japanese, and Chinese. For example, in an eye movement study, Kohsom and Gobet (1997) found that when reading Thai, native speakers’ reading rate is actually faster when spaces are artificially added to identify word boundaries. Winskel et al. (2009) also reported facilitative effect of interword spacing on reading speed and word recognition in Thai, but not the landing sites. Kasisopa et al. (2013) further found that Thai readers, as readers of spaced texts, tended to land their eyes near the word center, dependent upon the frequency of word boundary characters.

In terms of Japanese, Kajii et al. (2001) examined the landing-site distributions of the eyes during natural reading of Japanese scripts. Their results showed a clear preference for the eyes to land at the beginning rather than the center of the word, unlike in English. Further analysis for two- and three-character words indicated that the different landing-site distributions of the eye depend on the types of characters in the word. Specifically, the eyes prefer to land at the beginning of the word only when the initial character of the word is a Kanji character (i.e., Chinese). Sainio et al. (2007) also examined the role of interword spacing in reading Japanese. They found that interword spacing facilitated both word identification and eye guidance when reading a syllabic script (Hiragana), but not when the script contained ideographic characters (Kanji-Hiragana). They argued that in reading Hiragana, interword spacing serves as a segmentation cue, whereas spacing information in mixed Kanji-Hiragana texts is redundant because the Kanji characters are already visually salient by themselves and can serve as an effective segmentation cue. These findings suggested that salient demarcation of word boundaries is important in guiding readers’ eye movements during reading.

Results from studies in Chinese are somewhat less clear, and the main disagreement concerns whether eye movements are guided by characters or words (Liversedge et al., 2013). Yang and McConkie (1999), for instance, did not find a PVL for Chinese, as shown by quite evenly distributed eye fixations when reading unspaced text, and no word-based pattern of landing sites for the initial fixation location. Tsai and McConkie (2003) also proposed that eye guidance in reading Chinese is based on characters rather than words. However, more recently, contradictory findings have emerged. Yan et al. (2006) found an interaction effect between the frequency of the initial character in a word and the whole-word frequency, with word frequency modulating the effects of character frequency on total viewing times. This was taken as evidence of the words overriding individual characters. Consistent with this view, Rayner et al. (2007) simulated the eye movement behaviors of Chinese readers with the E-Z Reader model and confirmed that words were the unit of processing. In another study, Yan et al. (2010) examined whether Chinese readers first land their eyes at the word center or word beginning. Results showed that Chinese readers tended to land their eyes at the word center in single-fixation cases and at word beginning in multiple-fixation cases. The authors argued that readers of Chinese land their eyes on the word center if they successfully identify the word boundaries in parafoveal vision and on the word beginning if the segmentation failed. Findings from Li et al. (2011) also replicated these results. However, they did not take these findings as evidence for word-based saccade targeting in reading Chinese texts; alternatively, they propose that eye movement planning for Chinese readers may involve a combination of both character-based and word-based targeting. More recently, Zang et al. (2013) reported that the landing positions from adults and children were very similar when reading spaced and unspaced Chinese text. Their analyses showed that readers targeted their saccades similarly under spaced and unspaced conditions, and similar to Yan et al.’s (2010) finding, both Chinese adults and children targeted their saccades normally over the word with the PVL being close to the word center in single fixations, while in multiple fixation cases, the initial fixations were toward the word beginning. Shen et al. (2012) also replicated this finding with L2 learners. Taken together, these recent studies indicated that Chinese readers do not randomly select saccade targets; rather, the eye movement control in Chinese is word-based.

This then raised another question: Whether artificially inserting spaces between words to make the word boundaries visually salient in Chinese can benefit Chinese readers in terms of effective saccade targeting, reading speed and comprehension, and vocabulary acquisition. To address this question, research has further investigated whether adding spaces between words affects reading behaviors in Chinese. In an empirical study, Bai et al. (2008) presented Chinese texts in four conditions to native speakers: unspaced, appropriate spaces at word boundaries, appropriate spaces between all characters, and inappropriate spaces between characters resulting in apparent non-words. Results indicated that sentences written in word-spaced fashion were as easy to read for native Chinese speakers as the unspaced counterparts. However, spacing that created non-words and spaces between characters induced longer reading times. Their results indicate that the word rather than the character was the primary information unit for Chinese readers.

Researchers also used interword spacing as a pedagogical tool for children. For instance, Blythe et al. (2012) recorded the eye movements of 7- to 10-year-old children as they read new 2-character words that were embedded in sentences presented either in a normal, unspaced condition or in a word-spaced condition. The children were further tested on the new words embedded in a new (i.e., previously unread) sentence presented without spaces. Results showed that in the learning phase, children read the new words faster in the spaced sentences, and this facilitative effect was maintained in the test phase. The authors argued that it is because the spaces between words strengthened the connections between the two-character representations in children’s mental lexicon and thus facilitated word retrieval in the test phase. In short, recent research provides support for the view that words are important and have a psychological reality for Chinese readers.

In terms of L2 learners, not much research is available. In general, studies investigating whether interword spacing facilitates reading in Chinese among native speakers showed that adding interword spacing either did not influence their reading comprehension or speed (Liu et al., 1974; Everson, 1986; Bai et al., 2008) or hindered their reading performance (Bassetti, 2009; Bassetti and Masterson, 2012); however, a facilitation effect was found for L2 learners (e.g., Bai et al., 2013), which was modulated somewhat by the learners’ proficiency level (Everson, 1986; Bassetti, 2009; Yao, 2011; Shen et al., 2012) and native language. As one of the recent studies, Shen et al. (2012) used eye-tracking methodology and examined the relationship between spacing and word segmentation with four groups of non-native Chinese speakers (L1: English, Korean, Japanese and Thai) by using four types of spacing information: unspaced text, word-spaced text, character-spaced text, and nonword-spaced text. They found that the word-spaced text was the easiest for L2 learners to process, as indicated in the shortest total reading times and fixation durations, as well as lowest fixation counts and fewest regressions. The participants also found that the nonword-spaced and the character-spaced texts was the most disruptive, showing the longest reading times and more fixations and regressions. These effects, however, were independent of participants’ native languages, but decreased as participants’ proficiency level went up. Bai et al. (2013) also utilized eye-tracking to examine whether interword spacing facilitated vocabulary acquisition for L2 learners of Chinese. Following a similar design as Blythe et al. (2012), researchers found that participants read the new words faster in the spaced than in unspaced sentences, and this facilitative effect held in the subsequent test session. The authors attributed this benefit to the stronger connections made between the constituent characters due to the introduction of interword spaces. These findings, again, suggest that words have psychological reality for L2 learners of Chinese, and that pre-segmenting text into word units is beneficial.

Interestingly, besides the character form of Chinese texts that lack interword spaces, the Pinyin^2^ form of Chinese is word-spaced. However, it has been found that Chinese readers, both adults and school children, are much slower in reading the Pinyin form, compared with the reading materials in characters (Sun, 1993; Fu et al., 2002; Yan et al., 2008), which could be attributed to their lack of familiarity and practice with the Romanized form of Chinese, as well as the fact that Pinyin script provides no morphemic information, requiring longer time for readers to decode the meaning (Bassetti and Masterson, 2012). On the contrary, Pinyin has been found to be read faster by L2 learners of Chinese than characters (Light, 1976), and English learners of Chinese read Pinyin texts faster than native speakers (Bassetti, 2009). This could be because of L2 learners’ higher level of familiarity with the Pinyin form during their study of Chinese or their familiarity with reading word-spaced Romanized scripts.

The preceding review shows a paucity of studies examining L2 learners’ eye movements when reading spaced and unspaced Chinese texts. More importantly, all of the previous literature dealt with L2 learners’ reading performance when reading individual words or sentences, with no investigations extending beyond the sentence level. In response to the recent call for enhancing the ecological validity in eye tracking research (e.g., Elgort and Warren, 2014; Godfroid et al., 2018), longer reading materials can provide rich contextual information and better represent the natural reading context of readers. In addition, no previous studies have included Pinyin as a presentation condition to examine the potential effect of L2 learners’ native languages. The motivation for the current study, therefore, is to provide a descriptive account of the eye movements of CSL learners when reading spaced and unspaced passages in Chinese, with the hope to contribute to the theoretical accounts of eye movement control and word identification during L2 reading, and to shed light on the pedagogical issues of teaching Chinese orthography and second language reading development. This study was thus guided by the following research questions:

1. Will artificially inserting interword spacing in a Chinese text affect the reading behaviors of native speakers of Chinese, as measured by eye movement behaviors (e.g., fixation times and landing sites) and reading comprehension?
2. Will artificially inserting interword spacing in a Chinese text affect the reading behaviors of L2 learners of Chinese, as measured by their eye movement behaviors (e.g., fixation times and landing sites) and reading comprehension?
3. Are readers’ self-reported script and spacing preferences reflected in their eye movement records, with the more preferred script showing shorter reading times?

## Methods

### Participants

Twenty-four CSL learners who were enrolled at a Midwestern university in the United States were recruited for this study. All of the L2 speakers are native speakers of English. They were taking the third- or fourth-year Chinese class at the time of data collection and were regarded as intermediate learners based on a Chinese proficiency test targeting their reading skills from the American Council of the Teaching of Foreign Languages (ACTFL). Twenty native speakers (NS) of Chinese were also invited to participate as a control group.

Table 1 summarizes the demographic information of our participants. The NS control group included students (2 undergraduates, 17 graduate students, and 1 visiting scholar) enrolled at the university. All of them speak and read Mandarin Chinese and reported no hearing or vision problems. The L2 learners were either undergraduate or graduate students pursuing 16 different academic specializations. They had learned Chinese for an average of 4.3 years (ranging from 1.5 to 8 years, SD = 2.40), and reported to spend an average of 4.4 h (SD = 2.40) per week in reading Chinese. They also self-rated themselves as intermediate level on reading (M = 2.33, SD = 0.76) and writing (M = 2.04, SD = 0.55) in Chinese on a 4-point Likert scale (i.e., 1 = Beginning, 2 = Intermediate, 3 = Advanced, 4 = Near-native).

**Table 1:** Demographic information for participants.

Participants	Size	Gender	Age range, mean, SD (years)	Chinese proficiency
Native speakers (NS)	20	5 males 15 females	19–43, 28.6, 5.6	Native
Chinese as a second language learners (L2 learners)	24	11 males 13 females	18–29, 21, 2.27	Intermediate

### Apparatus

The participants’ eye movements were recorded with an EyeLink 1,000 desk-mounted eye tracker manufactured by SR Research Ltd.^1^, with a sampling rate of 1,000 Hz. The right eye was monitored. A chin-and-forehead rest was used to stabilize participants’ head movements during the recording. Stimuli were presented on a 19-inch DELL monitor at a viewing distance of approximately 50 cm. Reading materials were presented in Song font, size 21 (Yan et al., 2010; Zang et al., 2013) for the character conditions, and in regular Consolas font, size 18 for the pinyin condition, double spaced vertically. Each screen contained between seven to nine lines of texts, and each line contained 12–15 Chinese characters.

During the reading experiment, participants moved from one screen to the next by pressing a button, without being allowed to go back to the previous screens. A one-point drift correction was performed on each screen to minimize eye movement errors for reading multiple-line texts. The researcher calibrated the eye tracker four times in total (once for each passage, every 4 presentation screens) during the experiment, and conducted manual calibration check if eye movement errors occurred.

### Materials

#### Reading passages

Four narrative paragraphs were created with the vocabulary from the second-year Chinese textbook used at the L2 learners’ university. Each paragraph was approximately 350–400 characters in length, containing 14–17 sentences. Four presentation types were created for each paragraph: (1) normal unspaced text (US); (2) artificially word-based spaced text (WS); (3) nonword spaced text (NW); and (4) normal spaced text in Pinyin (PY). Word segmentation was based on Packard (2000) definition of a syntactic word as “the smallest form that can independently occur in a syntactic form class slot” (p: 12). The word segmentation was consulted about with eight Chinese native speakers who are linguistic majors at the same university. All the vocabulary from the textbooks was checked against the Outline of Chinese Standard Vocabulary and Chinese Characters Grading, and only words from Band A and Band B were selected in constructing the reading paragraphs. Eighty-one percent of the vocabulary corresponds to words from HSK (*Hanyu Shuiping Kaoshi*, a government-sponsored Chinese proficiency test) Levels 1 and 2. This would ensure the appropriate lexical coverage for L2 learners’ proficiency level. In addition, the experimental passages were carefully constructed to ensure that students were familiar with the topics (i.e., a trip to Yunnan, ways to keep fit and healthy, job hunting, and changes in Chinese cities).

#### Conditions

Four experimental conditions were generated for each passage (see Table 2 for examples for each condition).

**Table 2:** Example sentence for each condition.

Condition	Example sentences
Unspaced (US)	南京是一座美丽的城市。
Word-based Spaced (WS)	南京 是 一座 美丽的 城市。
Nonword Spaced (NW)	南 京是一 座美 丽的城 市。
Pinyin (PY)	Nánjīng shì yízuò měilìde chéngshì.
*Translation*	*Nanjing is a beautiful city.*

As seen in Table 2, the sentences took up different amounts of space, with the pinyin text being the most extended across the four experimental conditions. To ensure that each line contained the same amount of information, the layout of the passages was adjusted based on the pinyin text. In other words, the texts in the US, WS and NW conditions were forced to start from the next line based on the PY condition (i.e., the longest script).

A Latin-square design was adopted so that participants never read the same passage in the same condition, but were exposed to passages in all four conditions. To illustrate, if one participant read passage 1 presented in an unspaced fashion, passages 2, 3, and 4 in word-based spaced, nonword spaced, and pinyin condition, respectively, another participant would read passage 2 presented in an unspaced fashion, passages 3, 4, and 1 in word-based spaced, nonword spaced, and pinyin text, respectively. This manipulation resulted in 16 experimental lists in which participants read the scripts in a randomized order.

### Procedure

**Figure 1:**
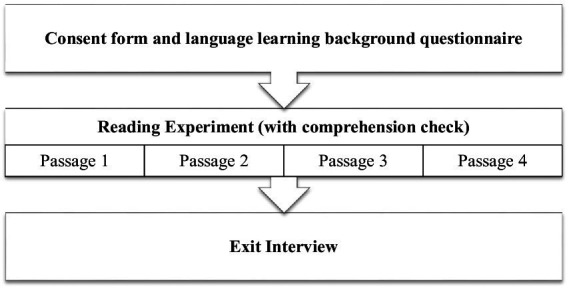
Experiment procedure.

During the experiment, the participants were tested individually in an eye tracking lab. The native and L2 speakers completed the same set of tasks. The experiment procedure is shown in Figure 1.

#### Language learning background questionnaire

To obtain information about the participants’ language learning experiences, an online language background questionnaire was administered. By responding to the background information questionnaire, which included information about their age, native languages, other foreign languages they had learned, length of exposure to Chinese, years of immersion in a Chinese-speaking environment, and the frequency of Chinese usage, participants indicated their consent to take part in the study. In particular, since the focus of this study concerned reading, participants were asked to provide information about their knowledge of other spaced scripts (for native speakers) and other unspaced scripts (for L2 learners). Participants also needed to indicate how often and how much they read both in their native language (i.e., English) and in Chinese.

#### Reading experiment

During the main experiment session, participants read six paragraphs in Chinese and answered comprehension questions for each paragraph. Among the six paragraphs, two were warm-up texts to familiarize the participants with the testing procedure. A set of paper-based comprehension questions was administered after each passage to measure participants’ comprehension of the reading passages as well as to encourage them to focus on text comprehension while reading. The comprehension questions were five True-or-False questions in which participants needed to judge the statements based on the paragraph contents they just read. Participants were informed beforehand that they would need to answer the comprehension questions after reading each passage. The questions were carefully constructed to contain text-specific information only. One point was assigned for correct answers, and zero was given for incorrect answers and the “Not sure” option.

#### Exit interview

After completing the reading task, each participant was interviewed with regard to the reading task that he or she just finished. Participants were asked: (1) Which version of the texts they found the most difficult? Which one was the easiest? Why? (2) What was the most difficult thing when reading an unspaced Chinese text? (3) Whether reading Chinese characters was easier than reading Pinyin? Or vice versa? (4) Whether they used any strategies when segmenting Chinese words while reading?

### Data analysis

For comprehension questions, non-parametric Friedman’s ANOVA analyses, with the presentation condition (US, WS, NW, PY) as the independent variable, were conducted for native speakers and L2 learners separately, given that the comprehension scores were not normally distributed.

For eye movement data, I conducted analyses on global measures for each presentation condition, namely, total passage reading time (i.e., the sum of all the fixation durations made on a passage); mean first fixation duration (i.e., averaged durations of the first fixation on a word), gaze duration (i.e., the sum of all fixations on a word prior to moving to another word), number of fixations (i.e., number of fixations made on each word), number of regressions (i.e., number of times a word was exited to an earlier part of the passage), and regression path duration (i.e., the sum of all fixations made from the first encounter with a word until the eyes move past the right boundary of a word, including any regressions to earlier parts of the passage). Those measures were analyzed as dependent variables. Normality tests showed that 95% of the measures were normally distributed, thus, 4 (presentation condition: US, WS, NW, PY) × 2 (participant group: native speakers and L2 learners) mixed-design ANOVAs for each eye movement measure were conducted.

Landing position analyses were also carried out to further investigate the PVL effect. Given that one-character words were primarily function words that are often skipped in reading, and that the number of three- and four-character words were relatively small, I conducted the landing distribution analyses solely on the two-character words. Additionally, because of the varying widths of the interest areas across the four conditions, I only included the normal unspaced and word-based spaced conditions for comparisons. Following Yan et al. (2010) and Zang et al. (2013) analyses of landing positions, I defined half of a character in a horizontal direction as a character zone. Therefore, a two-character Chinese word occupies four zones, ranging from 0 to 2 characters (0–0.5, 0.51–1, 1.01–1.5, 1.51–2, respectively), with a value of 1 indicating the middle of the word. Notably, following von der Malsburg and Angele (2017) who proposed that corrections of alpha-level should be applied in eye movement studies to control the Type I error (i.e., an inflated probability that the null hypothesis is incorrectly rejected), the alpha-levels of this study were adjusted by dividing .05 by the number comparisons made in both the global and local analyses.

## Results

### Comprehension questions

The overall reliability coefficient (Cronbach’s α) of the 20 comprehension questions for all participants was .83. The individual reliability coefficients for native speakers and L2 learners were .86 and .78, respectively. The descriptive statistics for the comprehension questions across four presentation conditions are shown in Table 3.

**Table 3:** Mean comprehension scores across the four presentation conditions (SD in parentheses).

Condition	Native speakers	L2 learners
Unspaced	4.40 (0.68)	4.54 (0.59)
Word-based spaced	4.60 (0.50)	4.25 (0.94)
Nonword spaced	4.45 (0.76)	4.29 (0.95)
Pinyin	4.75 (0.44)	4.25 (0.85)
Total	4.55 (0.84)	4.33 (0.61)

Overall, both participant groups demonstrated good understanding of the passages. Out of five questions for each passage, native speakers scored 4.55 out of 5 (SD = 0.84), and L2 learners scored 4.33 (SD = 0.61). Non-parametric Friedman tests showed that comprehension did not differ significantly across the four experimental conditions for either group (native speakers: χ^2^ = 3.05, *p* = 0.38; L2 learners: χ^2^ = 1.96, *p* = 0.58). The high comprehension rate in both groups may suggest a ceiling effect in the task; however, it helps to rule out the possibility that any spacing effect, if observed, could be attributed to the text comprehensibility.

### Eye movements

Table 4 summarizes the descriptive statistics for the eye movement measures across the four presentation conditions for both groups.

**Table 4:** Descriptive statistics for eye movement measures across the four presentation conditions (SD in parentheses).

Measures	Condition	Native speakers	L2 learners
Total passage reading time (s)	Unspaced	31.36 (10.27)	169.02 (47.02)
	Word-based spaced	34.45 (14.00)	159.51 (53.25)
	Nonword spaced	35.76 (13.08)	181.19 (53.14)
	Pinyin	173.96 (41.45)	162.34 (51.13)
First fixation duration (ms)	Unspaced	206 (23)	311 (47)
	Word-based spaced	199 (27)	306 (50)
	Nonword spaced	204 (26)	289 (40)
	Pinyin	266 (36)	274 (42)
Gaze duration (ms)	Unspaced	227 (32)	604 (124)
	Word-based spaced	218 (37)	591 (147)
	Nonword spaced	233 (41)	602 (115)
	Pinyin	572 (113)	515 (98)
Number of fixations	Unspaced	0.79 (0.22)	2.93 (0.80)
	Word-based spaced	0.89 (0.31)	2.78 (0.70)
	Nonword spaced	0.90 (0.30)	3.19 (0.75)
	Pinyin	3.53 (0.74)	3.20 (0.93)
Number of regressions	Unspaced	14.65 (6.71)	42.54 (18.08)
	Word-based spaced	15.05 (6.13)	37.00 (15.63)
	Nonword spaced	16.65 (6.29)	50.63 (21.89)
	Pinyin	47.60 (9.65)	46.21 (21.47)
Regression path duration (ms)	Unspaced	332 (107)	1,012 (319)
	Word-based spaced	324 (129)	906 (327)
	Nonword spaced	349 (80)	1,054 (317)
	Pinyin	1,007 (266)	957 (308)
Skip rate (%)	Unspaced	40.19 (10.57)	11.38 (6.3)
	Word-based spaced	10.63 (6.7)	13.27 (8.4)
	Nonword spaced	42.27 (9.3)	13.32 (9.3)
	Pinyin	36.39 (10.94)	10.14 (7.6)

Alpha level was set as.007.

For all the eye movement measures, there was a main effect of presentation condition (all Fs > 16.50, *p*s < 0.001, η_p_^2^ > 0.28, observed power = 1.00). There was also a significant difference between the two experimental groups (all Fs > 21.94, *p*s < 0.001, η_p_^2^ > 0.34, observed power = 1.00), suggesting that overall L2 learners spent longer time processing the texts, made more fixations and regressions, and skipped less than native speakers. The presentation by group interaction was also significant (all Fs > 32.88, *p*s < 0.001, η_p_^2^ > 0.44, observed power = 1.00), indicating that the spacing effect differed between the two participant groups.

For native speakers, the eye movement data showed a similar pattern for total reading times, first fixation duration, gaze duration, number of fixations, number of regressions, and regression path duration. Specifically, native speakers spent a significantly longer time processing the passages, made more fixations and regressions in the pinyin text than in the other three conditions (all *p*s < 0.001), and there were no differences between the normal unspaced, word-based spaced, and nonword spaced (all *p*s > 0.38) texts. However, for skip rate, native speakers made the fewest skips for word spaced text (M = 10.6%), and the number of skips was significantly lower than that in the other three conditions (pairwise comparisons, all *p*s < 0.001). The native speakers also skipped pinyin text (M = 42.3%) marginally less than the nonword spaced text (M = 36.5%, *p* = 0.014). The differences between the other pairs were not significant (all *p*s > 0.12). In general, interword spacing did not affect native speakers’ reading behaviors except that they skipped the word spaced text less. This suggests that adding interword spacing in Chinese texts may interrupt native speakers’ eye movements during natural reading.

Regarding the L2 group, the patterns were less clear. Table 5 summarizes the processing patterns for L2 learners.

**Table 5:** Processing patterns for L2 learners.

Measures	Patterns
Total passage reading time	nonword spaced > unspaced > pinyin > word spaced
First fixation duration	unspaced > word spaced > nonword spaced > pinyin
	* unspaced > nonword spaced
	* unspaced > pinyin
	* word spaced > nonword spaced
	* word spaced > pinyin
Gaze duration	Unspaced ≈ nonword spaced ≈ word spaced > pinyin
	* unspaced > pinyin
	* nonword spaced > pinyin
	* word spaced > pinyin
Number of fixations	Pinyin ≈ nonword spaced > unspaced > word spaced
	* nonword spaced > word spaced
Number of regressions	Nonword spaced > pinyin > unspaced > word spaced
	* nonword spaced > word spaced
Regression path duration	Nonword spaced > unspaced > pinyin > word spaced
	* nonword spaced > word spaced
Skip rate	Nonword spaced ≈ word spaced > unspaced > pinyin

(1) * indicates statistical difference for each comparison; (2) Alpha-level was set as .007.

Overall, L2 learners had the longest total times reading the nonword spaced text (M = 181,190 ms, SD = 53,139 ms), which was longer than the word-based spaced condition (M = 159,510 ms, SD = 53,249 ms, *p* = 0.023). There were no differences between the other conditions (*p*s > 0.11).

The first fixation duration was the longest for unspaced text (M = 311 ms, SD = 47 ms), and was significantly longer than the nonword spaced condition (*p* < 0.001) and the pinyin condition (*p* < 0.001). First fixation duration was also longer for the word-based spaced condition than the nonword spaced condition (*p* = 0.004) and the pinyin condition (*p* < 0.001). There were no differences between the other pairs (*p*s > 0.73).

Gaze duration was the shortest for the pinyin text (M = 515 ms, SD = 98 ms), and was marginally shorter than the other three conditions (all *p*s < 0.027), suggesting the relative processing ease of pinyin texts for L2 learners whose native language is alphabetic (e.g., English). There were no differences between the other conditions (*p*s = 1.00).

In terms of number of fixations, L2 learners made the fewest number of fixations on each word in word spaced text (M = 2.78, SD = 0.70), and fixations were significantly fewer than those in the nonword spaced condition (*p* < 0.001), and the pinyin condition (*p* = 0.002). There were no differences between the other condition pairs (*p*s > 0.08). Similarly, L2 learners also made the fewest regressions for word spaced text (M = 37.00, SD = 15.63), and regressions were significantly fewer than those in the nonword spaced condition (*p* < 0.001), and the pinyin condition (*p* = 0.007). There were no differences between the other pairs (*p*s > 0.21). These results suggest the processing ease with the word spaced texts for L2 learners because word boundaries in the word spaced condition are clearly marked, which reduces their processing burden in identifying words during reading.

Regression path duration was also the shortest for word spaced text (M = 906 ms, SD = 327 ms), and was marginally shorter than that in the normal unspaced condition (*p* = 0.023) and the nonword spaced condition (*p* = 0.009). Other comparisons were not significant (*p*s > 0.072). As a late eye movement measure that reflects the relatively late stages of processing, such as information reanalysis and discourse integration (Rayner, 2009), the regression path duration further suggests the processing ease with the word spaced texts because adding interword spaces reduces L2 readers’ cognitive load in reanalyzing information.

As for skip rate, there were no differences between the presentation conditions (all *p*s > 0.39) for L2 learners, suggesting that learners skipped similar amounts of words during reading regardless of the presentation format of the text.

To summarize, L2 learners’ eye movement data, though less systematic, showed that L2 learners had the most difficulties processing the nonword spaced texts, indicating the disruptive effect of nonword spacing on L2 readers’ reading process. More importantly, L2 learners spent more time reading the unspaced text than the word spaced text (although numerically for the total reading times), partially confirming the benefits of adding interword spacing into Chinese written text because interword spaces may facilitate readers to recover from processing difficulties (Rayner, 2009). In addition to these time-based measures, the number of fixations and regressions also suggested that nonword spaced text induced more difficulties than word spaced text; more importantly, L2 learners made numerically more fixations and regressions to the normal unspaced text than to the word spaced text. These eye movement events are often associated with global processing of the whole text. Early measures, such as first fixation durations, however, showed no differences between the normal unspaced and word spaced text. This could be because the vocabulary used to create experimental texts were quite familiar to L2 learners, which may have offset the potential facilitative spacing effect. Taken together, eye movement data from L2 learners showed that they overall benefited from reading word-based spaced text in Chinese, especially for discourse-level processing.

### Landing position analyses

In addition to the global eye movement measures, a set of local analyses were also conducted to investigate the PVL effect, which may provide a more fine-grained account of the spacing effect. For local analyses where a value of 1 indicates the middle of the word, a value smaller than 1 suggests that participants land their eyes on the first character, with smaller values meaning eyes landing more toward the word beginning; similarly, a value larger than 1 suggests that participants send their eyes to the second character, with larger values indicating eyes landing more toward the end of a word. Table 6 presents the mean landing positions for initial fixations, and the proportions of single fixations for native speakers and L2 learners when reading unspaced and word spaced texts.

**Table 6:** Mean landing positions (in characters) in unspaced and word-based spaced texts, with SDs provided in parentheses to indicate variability.

Measures	Native speakers		L2 learners	
	Unspaced	Spaced	Unspaced	Spaced
First fixation position	1.03 (0.09)	1.05 (0.76)	0.71 (0.11)	0.76 (0.12)
First fixation position in single fixations	1.05 (0.10)	1.05 (0.10)	0.94 (0.18)	0.96 (0.15)
First fixation position in multiple fixations	0.99 (0.20)	1.05 (0.16)	0.66 (0.09)	0.71 (0.12)
Proportion of single fixations (%)	73.62 (10.22)	74.33 (12.23)	17.95 (11.96)	20.27 (12.75)

Alpha level was set as.0125.

#### Mean landing positions for first fixations

There was a significant effect of spacing, F(1, 42) = 4.38, *p* = 0.042, η_p_^2^ = 0.09, observed power = 0.53. The landing positions were further into a word for word spaced text (M = 0.89, SD = 0.18) than for normal unspaced text (M = 0.86, SD = 0.19). That means, the initial landing positions were closer to the center of the word in spaced than in unspaced conditions. There was also a significant effect of participant group, F(1, 42) = 144.94, *p* < 0.001, η_p_^2^ = 0.78, observed power = 1.00; native speakers tended to fixate closer to the center of the word (M = 1.04, SD = 0.56) than L2 learners (M = 0.73, SD = 0.45). The interaction was not significant (*p* = 0.55), suggesting that the spacing effect held for both groups alike.

**Figure 2:**
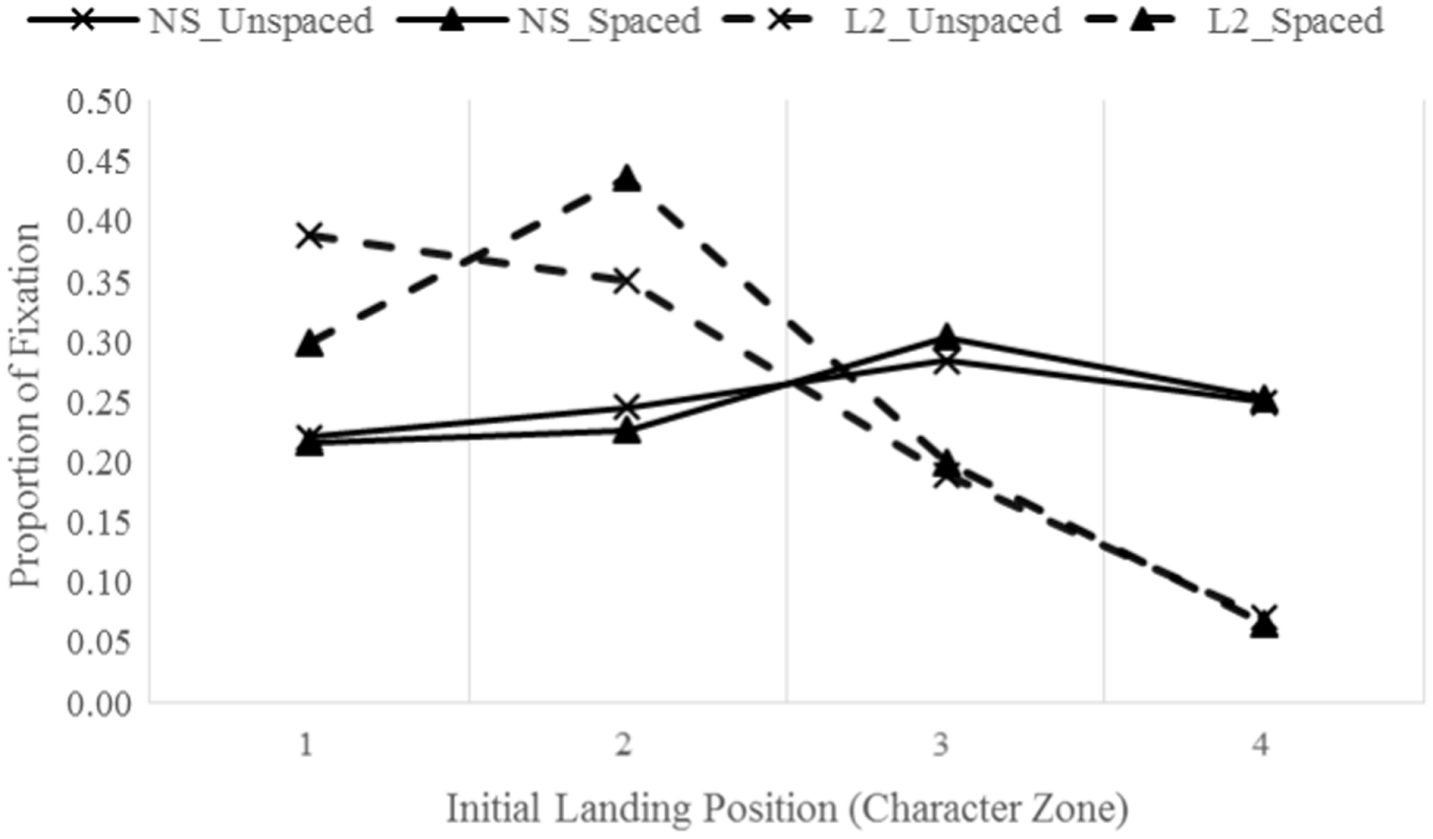
The distribution of landing positions of initial fixations.

A set of 2 (condition: normal unspaced and word-based spaced) × 2 (group: native speakers and L2 learners) × 4 (character zone: 1, 2, 3, 4) mixed-design ANOVAs were further conducted to examine the distribution of the initial fixations over each character zone of a word. The landing distribution data showed a significant effect of character zone (F(3, 126) = 41.17, *p* < 0.001, η_p_^2^ = 0.50, observed power = 1.00). The interaction between zone and group was significant, F(3, 126) = 64.81, *p* < 0.001, η_p_^2^ = 0.61, observed power = 1.00, and the interaction between condition and zone was also significant, F(3, 126) = 5.16, *p* = 0.002, η_p_^2^ = 0.11, observed power = 0.92. There was also a significant three-way interaction (F(3, 126) = 6.68, *p* < 0.001, η_p_^2^ = 0.14, observed power = 0.97). Figure 2 presents the distribution of the initial fixations over each character zone.

Further analyses revealed that native speakers were much more likely to fixate on zone 3 (M = 29.4%), less likely on zone 4 (M = 25.1%) and were least likely to fixate on zone 1 and 2 (M *=* 21.9, 23.6%, respectively, *p*s < 0.02). This pattern was identical across presentation conditions. L2 learners, on the other hand, were more likely to land on the first character (zones 1 and 2, M = 34.4, 39.3%, respectively), less likely on zone 3 (M = 19.4%, *p*s < 0.001), and least likely on zone 4 (M = 6.8%, *p*s < 0.001). This pattern held across conditions. However, as shown in Figure 2, when reading word-spaced text, L2 learners tended to target their saccades slightly closer to the word center.

Overall, native speakers landed further into a word than L2 learners. Additionally, Figure 2 shows that the saccade targeting of native speakers were almost identical in the spaced and unspaced texts. That is, native speakers were more likely to target their initial fixations to the word center of a two-character word regardless of whether the text was spaced or not. L2 learners, however, tended to direct their saccades to the beginning of a word, and more saccades were targeted to the word beginning for unspaced than spaced text. This is probably because L2 learners adopted a more conservative saccade strategy due to their limited proficiency in Chinese.

As Yan et al. (2010) noted, however, it is important to divide the data into single fixation (i.e., only one fixation made on a word) and multiple fixation (i.e., more than one fixation made on a word) cases because different saccade targeting patterns may occur for these two fixation events. Therefore, separate analyses were subsequently conducted for the two situations.

#### Mean landing positions for first fixations in single fixation events

The mean landing position for first fixations in single fixation cases, and the proportions of single fixations are shown in Table 6. For the mean initial fixation landing positions in single fixation cases, there was a significant effect of group, F(1, 42) = 12.02, *p* = 0.001, η_p_^2^ = 0.22, observed power = 0.92, indicating that native speakers performed differently than L2 learners. The effect of condition and the interaction between condition and group were not significant (all *p*s > 0.83), suggesting that the native-nonnative speakers differences held for both unspaced (M = 1.00) and spaced (M = 1.00) texts. Thus, spacing did not affect initial saccade targeting when readers made only one fixation on a word.

**Figure 3:**
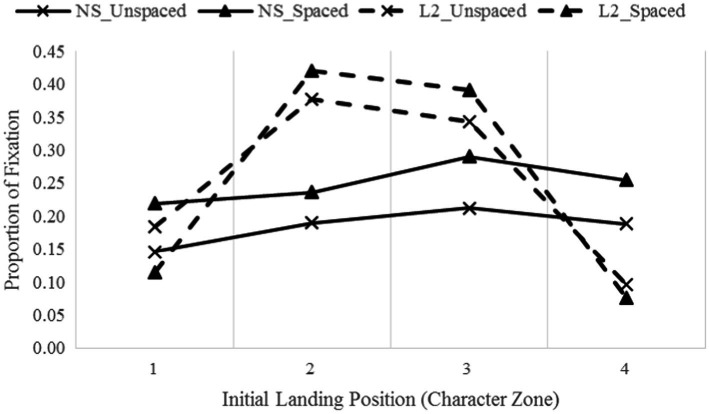
The distribution of landing positions in single fixation cases.

For the proportions of single fixation data (Figure 3), there was a significant effect of group, F(1, 42) = 279.33, *p* < 0.001, η_p_^2^ = 0.87, observed power = 1.00. Native speakers made more single fixations than L2 learners and made more single fixations (M = 74%) on a word than multiple fixations (M = 26%). The effect of presentation condition (*p* = 0.31) and the interaction between condition and group were not significant (*p* = 0.59), suggesting that readers made similar single fixations in unspaced and spaced texts.

The landing distribution analyses show that the effect of condition [F(3, 126) = 160.72, *p* < 0.001, η_p_^2^ = 0.79, observed power = 1.00] and the interaction between condition and group [F(3, 126) = 160.72, *p* < 0.001, η_p_^2^ = 0.79, observed power = 1.00] were both significant. The effect of character zone [F(3, 126) = 25.45, *p* < 0.001, η_p_^2^ = 0.38, observed power = 1.00] and the interaction between group and character zone [F(3, 126) = 18.40, *p* < 0.001, η_p_^2^ = 0.31, observed power = 1.00] were also significant. Other interactions were not so (all *p*s > 0.30). Further analyses show that native speakers tended to make more single fixations when reading spaced text (M = 25%) than unspaced text (M = 18.4%), and were slightly more likely to fixate on zone 3 and 4 (M = 25.1, 22.2%, respectively) than zone 1 (M = 18.2%, *p* = 0.015, 0.065, respectively). L2 learners, on the other hand, were more likely to land on the middle of the word (zones 2 and 3, M = 40, 36.7%, respectively), less likely on zone 1 (M = 14.9%, all *p*s < 0.001), and least likely on zone 4 (M = 0.8%, all *p*s < 0.001). This pattern held across the spaced and unspaced texts.

As indicated in Figure 3, native speakers landed further into a word than L2 learners in single fixation cases. That is, native speakers were more likely to target their fixations to the second character (zone 3, more toward the word center). Their saccade targeting was almost identical across presentation conditions, but they tended to make more single fixations when reading word-spaced text. L2 learners were also identical in terms of their saccade targeting pattern in spaced and unspaced text; they were more likely to target their initial saccades toward the word center regardless of whether the text was spaced or not.

#### Mean landing positions for first fixations in multiple fixations

The mean landing positions for first fixation in multiple fixation cases are shown in Table 6. Analyses of variance showed that the effect of presentation condition was marginal, F(1, 42) = 4.37, *p* = 0.043, η_p_^2^ = 0.09, observed power = 0.53. The landing positions were further into a word for word-spaced text (M = 0.88) than for unspaced text (M = 0.83). The effect of group was also significant, F(1, 42) = 88.95, *p* < 0.001, η_p_^2^ = 0.68, observed power = 1.00, with native speakers’ fixations landing closer to the word center (M = 1.02) than L2 learners’ (M = 0.69). The interaction between condition and group was not significant (*p* = 0.094).

**Figure 4:**
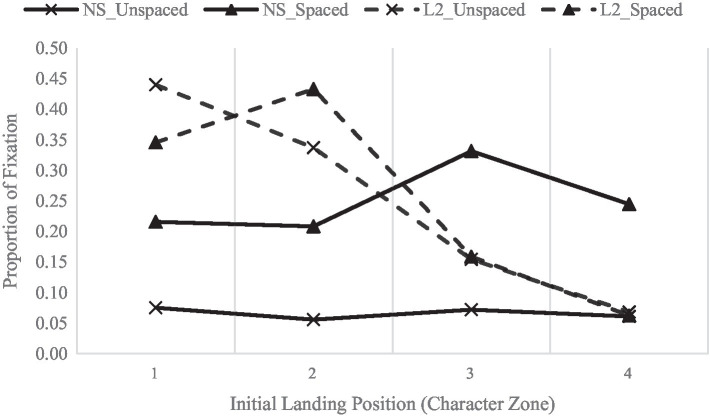
The distribution of landing positions in multiple fixation cases.

Figure 4 presents the landing distribution of initial fixations in multiple fixation cases. The landing distribution analyses show that the effects of presentation condition [F(1, 126) = 1251.63, *p* < 0.001, η_p_^2^ = 0.97, observed power = 1.00] and character zone [F(3, 126) = 43.85, *p* < 0.001, η_p_^2^ = 0.51, observed power = 1.00] were both significant. All the interactions were also significant (all Fs > 4.80, *p*s < 0.003).

Post-hoc tests showed that when reading spaced text, native speakers were more likely to fixate on word center than in the unspaced text (M = 25, 6.6%, respectively, *p* < 0.001). Saccades targeting pattern did not vary across character zones (all *p*s > 0.13, except that there was a marginal difference between zone 3 and zone 2, *p* = 0.014). L2 learners, on the other hand, were more likely to land on the first character (zones 1 and 2, M = 39.3, 38.5%, respectively), less likely on zone 3 (M = 15.7%, *p*s < 0.001), and least likely on zone 4 (M = 6.5%, *p*s < 0.001), regardless of the spacing condition.

Similar to previous discussions, native speakers generally landed further into a word than L2 learners. Additionally, Figure 4 shows that L2 learners were more likely to target their initial saccades to the beginning of words for unspaced text and toward the word center for spaced text. Native speakers, on the other hand, targeted more saccades to the second character (zone 3, more toward the word center) for spaced text.

## Discussion

In this eye-tracking study, I examined the eye movements of native speakers and CSL learners when they read normal unspaced texts, word-spaced texts, nonword-spaced texts and pinyin texts. The participants’ eye movement behaviors were compared across the presentation conditions to answer the main question: When learning an L2 that does not have interword spacing in written text, does inserting interword spaces benefit L2 learners’ online reading performance?

### Eye movements of native speakers

The first research question asks whether adding interword spacing in Chinese text affects the reading comprehension and eye movements of native speakers. The data, corroborating previous studies (Liu et al., 1974; Everson, 1986; Bai et al., 2008; Yao, 2011), showed that overall, interword spacing did not affect native speakers’ reading behaviors. This could be because of the ceiling effect caused by the reading materials. As mentioned earlier, the reading passages were constructed based on the second-year Chinese textbook, which was fairly easy for native speakers. The vocabulary selected also consisted of high-frequency words. Therefore, it is possible that native speakers easily identified the word boundaries based on their experience with those words, regardless of the presence of spaces (Yan et al., 2010). However, native speakers tended to skip the word-spaced text less frequently, suggesting that adding interword spacing in Chinese texts may interrupt native speakers’ eye movements during natural reading. In addition, native speakers experienced a hard time reading pinyin texts. This is not surprising because pinyin only denotes the pronunciation of Chinese characters but not their meaning, and there are many homophones in Chinese (Sun, 1993). That is, many characters share the same pronunciation but vary in visual forms and meanings. Hence, when reading the pinyin text, readers have to spell out the pinyin and are likely to search for the corresponding character from a set of competitors, based on the contextual meaning. This induced more processing difficulties and resulted in longer reading times, more fixations and regressions, and fewer skips. The exit interview data further supported this finding. Native speakers felt the pinyin most difficult and “disturbing” because first of all, it had been a long time (i.e., ever since Grade 1, age 7) for the native participants to read in pinyin. More importantly, when they read through the text, they had to go back to the previous parts to reparse the passage because of the limited visual information provided by pinyin.

In terms of the saccade landing positions, native speakers targeted their saccades very similarly under spaced and unspaced conditions, which replicated previous findings that the introduction of interword spacing did not affect native speakers’ eye guidance (e.g., Winskel et al., 2009; Yan et al., 2010; Li et al., 2011; Zang et al. (2013)). More specifically, similar to Yan et al. (2010), native speakers tended to target their saccades near the word center (i.e., PVL). However, slightly different from what Yan and colleagues have reported, this study found that native speakers tended to fixate on the beginning of the second character (close to the word center) regardless of the number of fixations made on a word. This difference arose probably because the reading materials were fairly easy for native speakers, and the word segmentation was easily accomplished during reading, resulting in a higher proportion of single fixations (i.e., 74%) on a word. Additionally, the proportion of targeting more toward the word center is numerically higher for the word-spaced than unspaced texts, indicating a nuanced advantage of adding interword spaces to improve the efficiency of word segmentation for native speakers.

### Eye movements of L2 learners

The second question asked whether adding interword spacing into a Chinese text affects the reading comprehension and eye movements of L2 learners. In terms of the comprehension questions, similar to native speakers, L2 learners did not show preferences for any presentation condition. In terms of the eye movements, the word-spaced texts seemed to be the easiest for L2 learners to process, especially when compared with the nonword-spaced text and pinyin text. In particular, L2 learners spent shorter total reading time in processing the passages, regressed less, and made fewer fixations on the word-based texts, which are measures that reflect the overall processing of the text. This result is partially consistent with Shen et al. (2012) findings. However, different from their results, the present study did not find significant differences between the normal unspaced text and word-spaced text. This may be due to the limited number of participants (*n* = 24) to achieve strong statistical power; however, another reasonable explanation could be that the L2 learners recruited from the advanced-level Chinese class had an average of 4.3 years of Chinese instruction, so they were quite used to reading unspaced texts. Additionally, the experimental passages were well within participants’ comprehension level, which may have modulated the differences between the unspaced and word-spaced texts. However, as 21 out of the 24 L2 learners indicated in the interviews, they felt the spaced texts quite strange at the first sight, but once they figured out that the spacing was based on words as in English, they felt it helpful for them to read faster later, which partly confirms the general advantage of adding interword spaces for L2 learners’ overall processing of a reading passage.

As for the landing sites, similar to what Shen et al. (2012) have found, L2 learners targeted their saccades almost identically under spaced and unspaced conditions. In particular, for single fixations, landing positions were normally distributed about the OVL of a word, while in multiple fixation cases, L2 learners tended to target their initial fixations toward the beginning of a word. This finding also implies that when L2 learners landed around the PVL of a word, they needed only one fixation on the word, whereas when their eyes landed toward the word beginning, they were much more likely to make multiple fixations on the word. More crucially, the analyses of landing distributions ruled out the possible explanation that the marginally reduced processing times for word spaced text for L2 learners were due to more effective saccade targeting toward the PVL because as shown in the eye movement data, L2 learners landed their eyes similarly regardless of spacing condition.

### Eye movements and word segmentation

The third question concerns if readers’ subjective script and spacing preferences were reflected in their eye movements, with the more preferred script generating less processing difficulty. Native speakers reported that they did not notice any differences in terms of the presentation mode except for the pinyin condition. They also claimed that the pinyin was considerably harder to read. This was also evident in their eye tracking data in that native speakers spent longer times, made more fixations and regressions, and skipped less when reading the pinyin text, whereas their eye movements were similar across the other three conditions. As for L2 learners, even though they reported the normal unspaced texts as the easiest one to read, their eye movement data showed that the unspaced condition was not processed more fluently than other conditions (excerpt for the non-word condition). This suggests that even though L2 learners were more familiar with the normal unspaced texts, the salient demarcation of word boundaries did unconsciously facilitate their retrieval of words and reading process. Additionally, L2 learners reported that the nonword spaced text was the most disruptive text to read, which was also reflected in their eye movement data that L2 learners spent considerably longer times, made more fixations and regressions when reading the arbitrarily spaced texts. Interestingly, learners also emphasized in the interviews that they could read faster with pinyin but comprehend better with characters. This was also evidenced in the eye tracking data that for L2 learners, total reading times, first fixation durations, gaze durations, and regression path durations were relatively shorter for the pinyin text; however, they made significantly more fixations and regressions, and skipped least for pinyin, compared to the character conditions. This indicates that, although pinyin is widely used as a helpful pedagogical tool for beginning L2 learners to read Chinese characters, it contains indirect information regarding word meaning, which induces reading difficulties and causes inefficient processing of meaning.

These findings are important in relation to our primary research question, that is, whether marking word boundaries in Chinese text could assist L2 learners. The beneficial effect of interword spacing was partially confirmed by the results from this study. This is probably because the interword spacing reduced the time needed to select a saccade target and removed the burden to segment character strings during reading. This implies that interword spacing could be a helpful pedagogical tool for CSL learners, especially for beginners, to identify words and strengthen the word-form associations during reading. Another implication from this study is that the word is the basic processing unit for L2 learners. Anecdotally, when asked about their strategies in segmenting Chinese written texts, all of my L2 participants indicated that they mentally segment Chinese written texts based on words. For instance, three of the learners said that they drew lines underneath a word or slashes between words; others stated that whenever they encountered processing difficulties during reading, they tended to resort to the vocabulary list to make sure certain characters make a word, and then went back to reading. In other words, even when a language does not have interword spacing in its writing system, the word is still the basic unit for native speakers and L2 learners to group the character strings. Once the connections between the constituent characters of a word have been strengthened by frequent input, L2 learners can retrieve words and read more efficiently, while the incorrect groupings of the character strings may alternatively disturb their reading performance. The most crucial implication from this study, however, is that L2 learners generally landed their eyes identically on the word regardless of the spacing condition. The different patterns produced were dependent upon whether only one fixation needed to be made on a word or not. This suggests that, echoing what previous studies have found for native speakers (e.g., Yan et al., 2010; Zang et al., 2013), L2 learners, especially intermediate learners who are experienced in reading Chinese texts, do not select saccades based on the visually salient spaces between words. Following this study and Shen et al. (2012) who found that L2 learners from various proficiency levels displayed different processing patterns, with lower-level learners benefiting more from interword spacing than higher-level learners, future studies can examine the facilitative effect of interword spacing on readers with varying levels of Chinese reading experiences (e.g., Shen et al., 2012) when reading passages or authentic reading materials, such as novels or stories.

## Conclusion

By examining the eye movements of native speakers and CSL learners, the present study extended evidence for the facilitative effect of interword spacing to the reading of connected passages for L2 readers. Given the relatively small sample size and the homogeneous native language background of the L2 participants in this single-experiment study, the results may not be generalizable to a broader CSL learners as a group; in addition, the experimental materials seemed quite easy for L2 learners, which may have caused a ceiling effect to demonstrate the advantage of adding interword spacing to Chinese texts clearly. However, this study was the one of the first research to incorporate passage reading in the eye movement literature for CSL learners, which better resembles L2 learners’ reading experience and contributes to the investigation of interword spacing effect on the overall processing behaviors when reading longer texts, which requires readers’ higher level of cognitive abilities to integrate information or understand the text on the discourse level, than individual sentences. Future eye-tracking studies are encouraged to use longer and more authentic Chinese texts to further investigate the L2 processing mechanisms of Chinese words while reading with a larger number of CSL learners from various native language background. Lastly, it should be noted that adding interword spaces is not the only one option to facilitate beginning readers of Chinese, including both native and L2 readers. CSL teachers are also recommended to try with alternating-color words in Chinese texts with beginning readers (e.g., Perea and Wang, 2017; Zhou et al., 2020; Pan et al., 2021), which has the advantage of keeping the characteristics of Chinese writing system unchanged and providing learners with more natural reading environment.

## Data availability statement

The raw data supporting the conclusions of this article will be made available by the authors, without undue reservation.

## Ethics statement

The studies involving human participants were reviewed and approved by Michigan State University Institutional Review Board. The patients/participants provided their written informed consent to participate in this study.

## Author contributions

YC contributed to conception and design of the study, conducted the eye-tracking study, performed the statistical analysis, wrote, revised, and approved the submitted manuscript.

## Funding

This research was funded by the Fundamental Research Funds for the Central Universities (no. E2E41701) to the author.

## Conflict of interest

The author declares that the research was conducted in the absence of any commercial or financial relationships that could be construed as a potential conflict of interest.

## Publisher’s note

All claims expressed in this article are solely those of the authors and do not necessarily represent those of their affiliated organizations, or those of the publisher, the editors and the reviewers. Any product that may be evaluated in this article, or claim that may be made by its manufacturer, is not guaranteed or endorsed by the publisher.

## Footnotes

^2^Pinyin is a Romanization form to denote the pronunciation of Chinese characters, which is used widely as a pedagogical tool for beginning learners of Chinese, such as Chinese children and L2 learners of Chinese.

^1^ www.SR-research.com
